# A Comprehensive Texture Segmentation Framework for Segmentation of Capillary Non-Perfusion Regions in Fundus Fluorescein Angiograms

**DOI:** 10.1371/journal.pone.0093624

**Published:** 2014-04-18

**Authors:** Yalin Zheng, Man Ting Kwong, Ian J. C. MacCormick, Nicholas A. V. Beare, Simon P. Harding

**Affiliations:** 1 Department of Eye and Vision Science, University of Liverpool, Liverpool, United Kingdom; 2 St. Paul's Eye Unit, Royal Liverpool University Hospital, Liverpool, United Kingdom; 3 Malawi-Liverpool-Wellcome Trust Clinical Research Programme, Blantyre, Malawi; Charité University Medicine Berlin, Germany

## Abstract

Capillary non-perfusion (CNP) in the retina is a characteristic feature used in the management of a wide range of retinal diseases. There is no well-established computation tool for assessing the extent of CNP. We propose a novel texture segmentation framework to address this problem. This framework comprises three major steps: pre-processing, unsupervised total variation texture segmentation, and supervised segmentation. It employs a state-of-the-art multiphase total variation texture segmentation model which is enhanced by new kernel based region terms. The model can be applied to texture and intensity-based multiphase problems. A supervised segmentation step allows the framework to take expert knowledge into account, an AdaBoost classifier with weighted cost coefficient is chosen to tackle imbalanced data classification problems. To demonstrate its effectiveness, we applied this framework to 48 images from malarial retinopathy and 10 images from ischemic diabetic maculopathy. The performance of segmentation is satisfactory when compared to a reference standard of manual delineations: accuracy, sensitivity and specificity are 89.0%, 73.0%, and 90.8% respectively for the malarial retinopathy dataset and 80.8%, 70.6%, and 82.1% respectively for the diabetic maculopathy dataset. In terms of region-wise analysis, this method achieved an accuracy of 76.3% (45 out of 59 regions) for the malarial retinopathy dataset and 73.9% (17 out of 26 regions) for the diabetic maculopathy dataset. This comprehensive segmentation framework can quantify capillary non-perfusion in retinopathy from two distinct etiologies, and has the potential to be adopted for wider applications.

## Introduction

The vascular network supplying the inner retina is visible to examination and imaging, and consists of branches from the central retinal artery and vein. The retinal vasculature is finely tuned to meet high physiological demands, and dysfunction can result from several diseases. In diabetic retinopathy vascular damage leads to capillary non-perfusion (CNP), focal inner retinal ischemia, and neovascularization [1], [2]. Diabetic retinopathy is the commonest complication of diabetes, and the commonest cause of blindness in people of working age [2]. CNP also occurs in cerebral malaria [3], and in this disease impaired retinal perfusion is thought to be caused by sequestration of parasitized erythrocytes in retinal vessels [4]. Retinopathy is highly clinically significant in both diabetes and cerebral malaria. Malarial retinopathy is the best clinical diagnostic indicator of cerebral malaria in children, which is a common cause of death and disability in developing countries [5]. CNP is also seen in other occlusive and/or inflammatory conditions including central and branch retinal vein occlusion [6], sickle retinopathy [7] and Eales disease (tuberculosis) [8]. More subtle reductions in peri-foveal capillary density have been observed in association with hypertension [9].

The capillary network of the inner retina can be observed using fundus fluorescein angiogram (FFA), which provides information about microvessel structure and function, and is a standard investigation for many eye diseases. Abnormalities may include vascular blockage (arteriolar or venular occlusion, or CNP) or leakage of fluorescein from vessels indicating breakdown of the blood-retina barrier [6]. The spectrum of CNP includes subtle changes making accurate measurement difficult. It is however important as it may provide a useful marker and quantification of retinal ischaemic damage. Insofar as retinal vascular injury reflects systemic pathology, quantification of retinal changes may provide surrogate information about other microvasculatures, none of which are as accessible to examination and imaging [10]. Automated CNP segmentation therefore has several potential clinical uses in common sight-threatening and life-threatening diseases. Automated detection of CNP regions, however, is relatively undeveloped as yet [11], [12], [13]. Due to loss of capillaries, CNP is characterized by a change in texture rather than intensity compared to adjacent retinal tissue. This means that a texture segmentation framework, as described in Section 2, is an ideal method to address this problem.

Texture (or repeated patterns of intensities) has long been used in image processing and computer vision tasks since it is present in most objects of interest, and entails statistical or contextual information of image pixels which are best characterized by randomness, regularity (or periodicity), directionality and orientation [14], [15]. An interpretation of texture as the variation of data at scales smaller than the scale of interest [10], [16] allows extraction of pixel features within the context of surrounding pixels, and provides a powerful global representation of complex images. However, segmentation of texture images is intrinsically more challenging than intensity, since it depends on pixel patterns that are difficult to capture or define.

Existing texture segmentation strategies can be grouped into two categories: supervised and unsupervised. The former normally involve feature selection and/or extraction, along with acquisition of a training dataset to generate and train the classifier to perform segmentation on new unseen images [17]. Unsupervised segmentation can be achieved by different approaches [18], [19], [20], [21], [22], [23], [24], [25]. In spite of significant efforts unsupervised strategies have not yet been successfully employed for real world applications, since it is difficult to be application specific without considering domain knowledge. Medical images pose particular challenges since segmentation has to be highly accurate and the images are often noisy. In addition segmentation accuracy must be evaluated in the context of clinical practice, since adjacent structures may appear to be similar but arise from different pathologies and have highly contrasting clinical significance. The essential need for domain knowledge can be met by employing a supervised segmentation strategy where expert knowledge is used to refine segmentation and achieve good performance.

In this paper we describe and evaluate a comprehensive texture segmentation framework for the segmentation of CNP in FFA images. This novel framework combines unsupervised approaches highlighted by the state-of-the-art total variation segmentation models with supervised ensemble classification techniques. The remainder of this paper is organized as follows. In Section Methodology, the proposed texture-based segmentation framework will be described in detail, especially highlighting a new region term formula employing kernel techniques. The dataset and the evaluation strategies will be introduced in Section Experimental Methods. Experimental results are presented in Section Results. Section Discussion and Conclusions concludes the paper.

## Methodology

**Figure 1:**
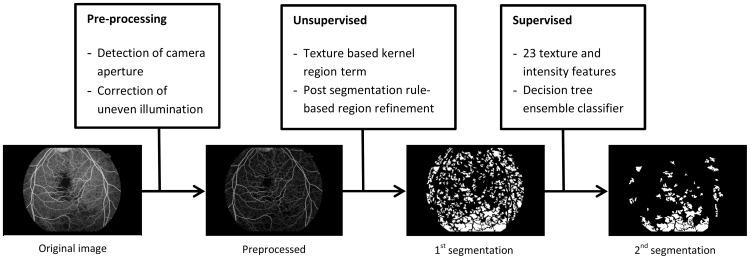
Flow diagram of the proposed segmentation framework for the retinal capillary non-perfusion segmentation on a fundus fluorescein angiogram image.

In this section we will describe the proposed texture segmentation framework in detail. Our framework uses a novel unsupervised texture segmentation technique that employs a total variation energy minimization algorithm. The segmentation results of this algorithm then become candidate regions for further refinement by a supervised ensemble classifier, which is trained with a set of textural features. Therefore the framework consists of three major steps, pre-processing and the two segmentation steps, as is demonstrated by the diagram in Fig. 1.

### A. Pre-processing/Pre-segmentation

In most image processing applications, pre-processing is a standard step to improve the image quality and/or to determine the region of interest. It generally involves one or more of the three main approaches: image denoising, deblurring, and enhancement. Image enhancement is the most widely used [26], [27]. In clinical retinal photography for instance, uneven illumination during fundus photography often leads to variations in image intensity that must be corrected by image processing. These operations usually begin with detecting regions of interest, which is the camera aperture, also known as the field of view (FOV) of the fundus image. In this work we propose two major pre-processing steps for CNP segmentation: FOV detection and uneven illumination correction.

**Figure 2:**
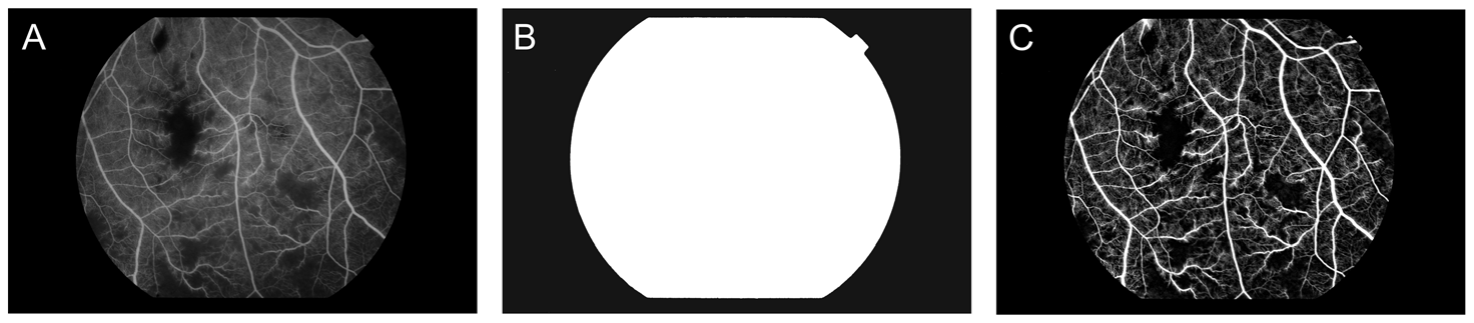
Preprocessing: (from left to right) Original image (A), field of view mask (B) and enhanced image after uneven illumination correction (C).

In a typical FFA image (e.g. Fig. 2A) the retinal FOV is surrounded by a black background. This background is removed using a mask, generated by thresholding and morphological operations on the image [26]. Thresholding distinguishes the dark background outside the camera aperture (or FOV) where the value of optimal threshold is determined by the Otsu approach. An opening operation is used to remove some isolated small regions in the background due to noise while a closing operation is necessary to remove any artifacts of the mask in the FOV. These can result from hemorrhages since their dark appearance on FFA is similar to the aperture background. A mask of Fig. 2A is shown in Fig. 2B.

The quality of fundus images may vary, due to factors such as eye movement, media opacity, small pupil size, camera misalignment and poor focus. Image enhancement is therefore essential [28]. Low frequency artifact, uneven illumination, poor contrast and blurring are common problems [29], [30], [31]. After the mask generation a top hat filter (structural element size: 50) is used to reduce low frequency noise. The following Fig. 2C demonstrates the resulting enhanced image.

### B. Texture-based Segmentation

This section is dedicated to providing mathematical details of the variational texture segmentation models which is the central piece of our segmentation framework. In recent years total variational models have become popular in dealing with many different image analysis problems and image segmentation in particular. Image segmentation can be formulated as an energy minimization problem that can be optimized using efficient solvers. We have previously published a general multiphase model that can deal with both texture and non-texture segmentation problems [32]. This model can deal with multiphase segmentation problems, that is, in an image where there are more than two categories of patterns or regions. Conventional segmentation is a special case of this model. We will first describe the multiphase modes in general in Subsection Multiphase Model and then briefly describe how this can be simplified for two-phase model in Subsection Two-phase Model. Finally, important extensions of region term to the original model that allow neighborhood information to be incorporated during the segmentation are described in Subsection Region Terms.

#### 1) Multiphase Model

Let *Ω* be a bounded open subset of 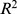 and 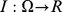 be a given two-dimensional (2D) grayscale image. The aim of segmentation is to partition 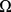 into N regions 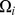, 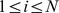 where 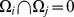, and 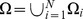. After segmentation, boundaries of all the phases 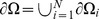 where 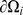 is the boundary for region 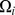, 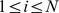.

According to [24], [25], a general multiphase segmentation model for both texture and non-texture segmentation problems can be formulated to minimize the following energy,
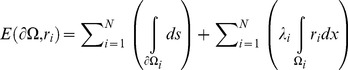
(1)The first term concerns the smoothness of region boundaries. The second term is known as the region term which enforces the similarity measurement between different regions by 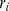 and 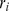 can vary depending on the problem domain and will be discussed in detail later this section. (1) can be reformulated as a total variation framework as the following
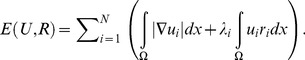
(2)Subject to 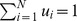 and 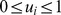, 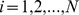. Where 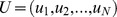 and 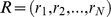. 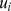 is the fuzzy membership values of phase 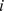 for each pixel while 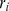 is the region term for phase 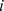. 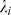 is the regularization weighting factor, by using different 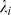 values for different phases, one can specify the relative influence of each individual phase. Here for simplicity we limit our discussion to the case where 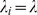. However, it would be straightforward to adapt the current formulation to cases with different values of 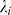.

In order to achieve a fast solution, an auxiliary variable 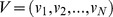 was introduced, resulting
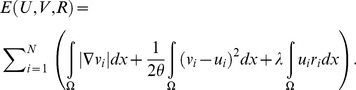
(3)Here the convex form 
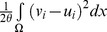
 is adopted to force 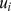 and 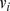 to be as close to each other as possible, where 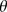 is a small positive number. Following the principle of Euler-Lagrangian optimization, the constraint 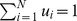 was explicitly included in (3) as
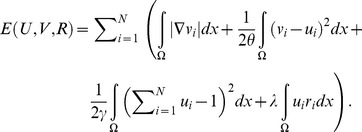
(4)(4) can be elegantly minimized by Chambolle's classic algorithm [33], more specifically, this can be done by alternately minimizing two energies, shown as follows,

Step 1: Solve 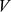


First, we consider the energy minimization problem below as solving 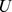 with fixed 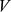 and 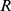.
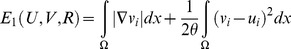
(5)This energy is tractable under Chambolle's fast projection program as follows,
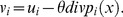
(6)Here 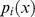 can be solved by a fixed point method by initializing 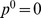


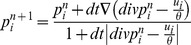
(7)where 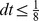 is the time step. See [33] for more details.

Step 2: Update 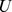


Secondly, by fixing 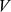 and 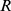 we solve 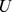 through considering the energy minimization problem below
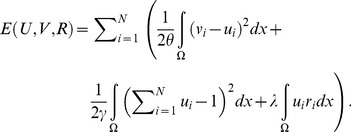
(8)

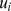
 can be directly derived as follows,
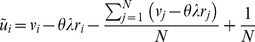
(9)After projection 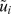 onto 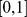, the solution for 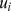 is obtained by the following,
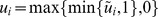
(10)


Step 3: Computation of Region Term R

As we remarked above, the model described here has the flexibility to tackle both texture and non-texture problems. In the above formulation 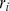 is a generalized expression of difference between two region terms, which should be specified for a particular segmentation problem. Several popular region terms as well as our new one will be detailed in Subsection Region Terms.

In summary the algorithm for multiphase segmentation can be stated in the following steps:

1. Initialization of 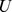 and 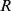.
2. Iteration
  1. Update 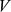 by (6);
  2. Update 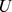 by (10);
  3. Estimate 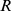 according to the specific region term chosen;
3. Terminate when 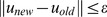, where 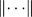 denotes the Euclidean distance and 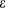 is a small positive number.

#### 2) Two-phase Model

For the sake of simplicity we considered the segmentation of CNP as a two-phase problem 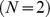. Compared to the multiphase case, now 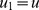 and 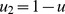, which explicitly enforces the constraint 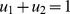. This can reduce the computational cost required by multiphase formulations. For completeness we give the major steps below.

Step 1: Initialization of 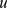 and 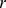.

Step 2: Iteration

- Update 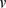 by 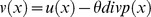, where 
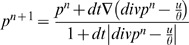
.
- Update 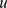 by 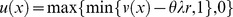

- Compute 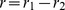, 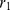 and 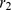 can be derived according to region term formulae as appropriate (see the next Subsection).

Step 3: Terminate when 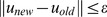.

#### 3) Region Terms

In this section three well-known region terms are described first followed by description of our new kernel-based region term for texture segmentation. Notably, other variants of region term definitions (i.e. [24]) can also be adopted by this model, reflecting the flexibility of the proposed multiphase model.

If 
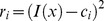
, 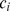 is the mean intensity of region 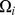. This model is the multiphase implementation of the celebrated *Chan-Vese (CV)* [34]. This is very effective in dealing with piecewise constant intensity segmentation problems.

If we consider the mutual information based region term as 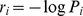, then the model can be used for texture segmentation. In this formulation 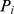 is the probability distribution function of the intensity of region 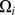. In [20], 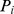 is assumed to be a Gaussian distribution for ease of computation. In real world problems a parametric model does not always hold, thus a non-parametric strategy was proposed by Kim et al [21] where the probability distribution 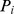 can be estimated by the Parzen window technique [35]. That is, if 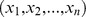 is an independent and identically distributed sample with an unknown density *f*, its kernel density estimator is
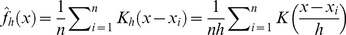
(11)Here 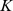 is the kernel and the bandwidth 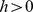 is a smoothing parameter. In our problem a Gaussian kernel with mean of zero and variance of one is used. Readers are referred to [35] for further details.

Ni *et al.* proposed a global convex minimization model that employed Wasserstein distance as region term [22]. Wasserstein distance is a metric used in optimal transport problems to measure the optimal transport cost. The Wasserstein distance with exponent 1 is defined as
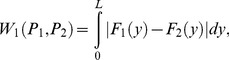
(12)Where 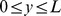, 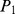 and 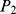 are histograms and their corresponding cumulative distribution are 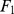 and 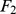 respectively.

The aforementioned region terms play essential roles in the segmentation models and here we propose a new way to formulate a region term for improved performance, based on the following observations. When looked closely at the region term, each pixel 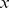 has a contribution of 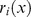 to the total error of the minimization problem. If a pixel is corrupted with noise or other artifacts, as will the contribution of 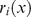. Intuitively, this problem can be alleviated by considering the information in its neighboring region surrounding the pixel 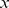. More specifically we propose the use of kernels to take into account contribution of each pixel 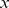 with the kernel window centered at itself.

Let 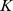 be a symmetrical positive and smooth window with dimensions of 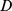. So the error of each pixel becomes 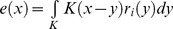, which is now dependent on the position. This error can be seen as a weighted average of error within the window centered at the point 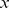. In many applications Gaussian kernel 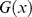 has been widely used for scale analysis, as its standard deviation can provide the model with an intrinsic spatial scale. If the window size of the kernel becomes one, it will become the conventional region term. In particular, the error 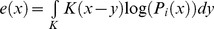 can be further written as 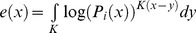. Considering its discrete nature of the problem the error becomes 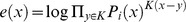, which is the weighted average of log-likelihood function over the window 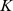. We observed that large window size will reduce the error of misclassification since it allows more regional statistical information to be considered, and to capture features at a larger scale. The use of inappropriately large windows may lead to inaccurate boundary detection, due to the smoothing nature of any kernel in use. There is a trade-off between window size and segmentation accuracy. In practice window size is expected to be empirically chosen for specific problem application. Note that Choy et al [24] has adopted an average window strategy for similar purpose which can be seen as a special case of kernel analysis here. Li et al has used the concept of kernel for intensity-based segmentation problems [36].

In the numerical implementation, the final region term can be computed as
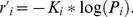
(13)* denotes convolution operation. 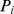 can be estimated from the Parzen window approach as before. If the kernels for each phase are the same, the above formulae can be further simplified.

**Figure 3:**
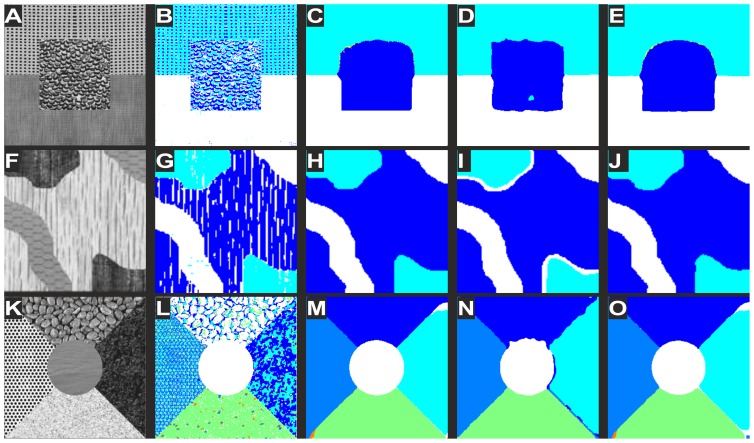
Illustration of multiphase texture segmentations. Difference between the aforementioned total variation models only differs in their region term. Top row: three-phase problem; Bottom row: five phase problem. Each row, in situ left to right: original image, segmentation results using region terms from CV model, local histogram, mutual information, and the proposed mutual information kernel.

**Figure 4:**
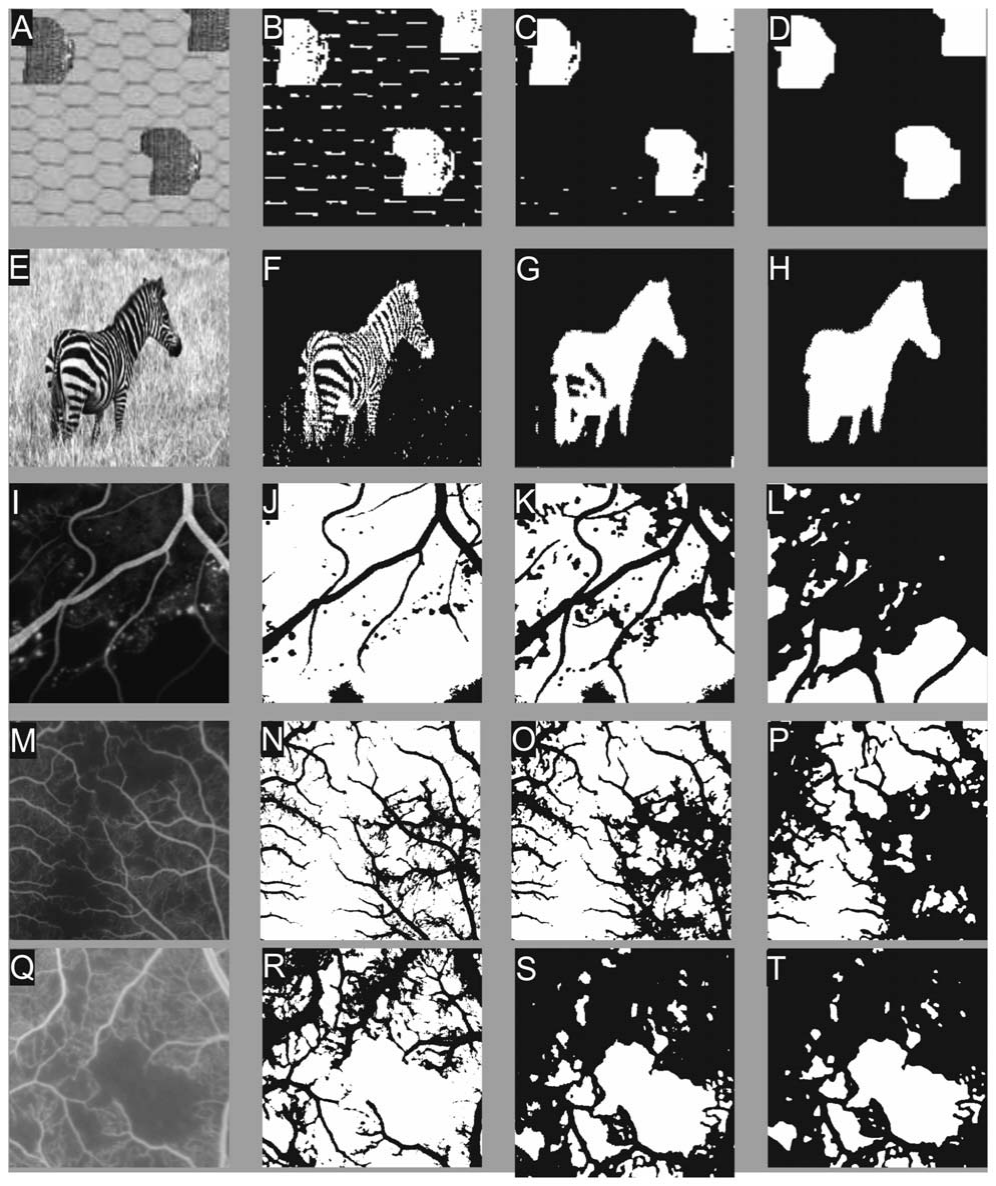
Results of two-phase segmentation with different region terms. Left: original image; middle left: results of CV model. Middle right: results of mutual information model; right: results of our new models. Images from top to bottom: synthetic, natural scene and CNP images (image I) from diabetic retinopathy followed by 2 images from malarial retinopathy (M and Q)).

Some examples of three and five phase problems individually solved by the CV model, mutual information region term, local histogram region term and kernel mutual information region term are shown in Fig. 3 to demonstrate the abilities and limitations of each model. The CV model fails to segment texture as it lacks a region term that can describe contextual information. The local histogram model performs best in the three phase problem (Fig. 3A–E), while in the five phase problems (Fig. 3F–J, K–O) it cannot segment boundaries correctly. The boundary effect of the local histogram model is caused by the window required for histogram information. Mutual information and kernel mutual information presents little or no boundary effects. We tested our model on a synthetic image, a natural scene, and three cropped FFA images with CNP regions one from ischemic diabetic maculopathy and two from malarial retinopathy. Each image is processed with the CV model, Zhu's mutual information model and our texture-based model, presented from left to right in Fig. 4. Values for 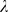 are chosen in each case for the most effective segmentation results. Our texture-based model can distinguish textures most effectively, demonstrated by these example images, while the CV model fails as expected because of the lack of region contextual information.

Use of local histograms was judged to be impractical in terms of data storage and speed. We therefore adopted the new kernel based region term 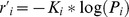 for this work.

### C. Supervised Segmentation

This multiphase texture segmentation model appears to be robust and accurate [32]. Regardless of how powerful an unsupervised classification method is, however, reference standard classification is often subjective to the clinician and so correct classification of every single pixel of clinical images is unrealistic. Consider retinal CNP: the algorithm segments all regions with similar textures as CNP. However, regional anatomy and image characteristics are not uniform, and so this may not be correct for all retinal regions. For example, the foveal avascular zone (FAZ) is a normal anatomical feature, but looks the same as an area of CNP, which represents pathology. Images of the retinal periphery may magnify inter-vascular spaces, and suggest CNP where none exists. Expert knowledge is needed to solve specific problems, and can be incorporated using a supervised method, which provides the flexibility to incorporate the domain knowledge specific to the application.

A set of clinically defined rules (defined by IJCM & NAVB) is used to eliminate CNP areas too small to be meaningful before the supervised segmentation step. More specifically, regions with major axis length <50 pixels are removed; this dimension approximates the diameter of a major vessel crossing the margin of the optic disc (∼125 µm). Conversion to pixels was estimated by considering FOV size and resolution. The major axis length of a region is the largest axis of the ellipse sharing the same normalized second central moments (Definition from Matlab [Mathworks, Natick, MA]). Secondly, morphological opening is performed to ‘tidy up’ elongated regions with width smaller than 125 µm such as those due to border effect. Since few test images included the FAZ this was removed manually when present. Automated FAZ detection is possible and will be implemented in future work [37].

For supervised segmentation, various classification techniques are currently available, such as artificial neural networks, support vector machine, decision trees, and the choice of classifier is dependent on the complexity of that specific application and the nature of the data. We have adopted the AdaBoost classifier, and used a set of FFA images for the training required in this supervised technique. The adaptive boosting (Adaboost [38]) classifier was chosen for false positive removal. Adaboost works by building a stronger and more powerful classifier from lots of smaller weak classifiers. We used a decision tree as the weak classifier [38]. The weak classifiers are generated sequentially in order to decrease the estimation error of the previous weak classifier [26]. Good sensitivity and specificity are achieved, despite imbalanced data from false positive detection of CNP, by using different weights for two classes. An ensemble classifier can be trained and used for prediction on new images. Care must be taken when generating the ensemble classifier to avoid overfitting problems.

The Adaboost classifier is trained by a training dataset consisting of manual annotations of CNP marked by a retinal specialist for “ground truth” (described in the next section). For each image a total of 23 features of each region detected in the previous stages are extracted. Features can be split into two main categories: intensity features, and texture features.

#### Intensity features (#1–11)

Features 1–5 are overall intensity (sum of all pixel intensities), mean, standard deviation, minimum and maximum pixel intensity of each region. Features 6–10 consider edge intensity information of each region, including overall edge intensity (sum of edge pixel intensities), edge mean intensity, standard deviation of edge intensity, minimum edge intensity and maximum edge intensity. The edge of each region is firstly determined by obtaining the perimeter of each region. The operations performed for features 1–5 are then applied to edge pixels (instead of the entire image) in order to generate features 6–10. The final intensity based feature, mass displacement, is defined as the distance between the center of gravity of the gray level intensities and the binary intensities of the region.

#### Texture features (#12–23)

The well-known Haralick features are used as texture based features, where the region pixels are first transformed into the co-occurrence matrix, and then 12 statistical features are calculated from it. These include: angular second moment (sum of the co-occurrence matrix squared), contrast (contrast weight), correlation (Pearson's correlation coefficient, covariance of horizontal and the vertical sum of the co-occurrence matrix divided by the product of their standard deviation), variance (square of standard deviation), inverse difference moment (measures homogeneity, co-occurrence matrix times by the inverse of contrast weight), entropy, sum of average of the co-occurrence matrix, sum of variance of the co-occurrence matrix, sum of entropy of the co-occurrence matrix, variance difference, entropy difference and two forms of information measure of correlation. These features are described by Haralick et al. [39].

## Experimental Methods

In this section we will describe the dataset used, the evaluation criteria, and the tests performed to evaluate the effects of various parameters on segmentation.

### A. Dataset

The proposed automated CNP segmentation framework was evaluated using two datasets: images from malarial retinopathy and ischemic diabetic maculopathy respectively.

The malarial retinopathy dataset comprises forty-eight FFA image frames (one per sequence)that were collected from children in Malawi. All images were taken with 50 degree FOV at a size of 3008×1960 pixel using a fundus camera (TRC-50 EX, Topcon, Tokyo, Japan) and were graded by visual inspection to ensure the image quality was adequate for image processing. One ophthalmologist (IJCM) manually selected images with CNP in an appropriate FFA phase. Early phase images display normal capillary filling, which may look like CNP, while late phase images may have vessel leakage that obscures genuine CNP. The ophthalmologist also manually annotated all CNP regions with estimated maximal linear diameter of >125 µm and marked the FAZ as a separate region. Manual annotation of CNP formed our reference standard.

The diabetic maculopathy dataset comprises ten FFA image frames collected from patients with diabetes. These images were taken using an HRA2 scanning laser ophthalmoscope (Heidelberg Engineering, Germany). These images were graded and manually annotated following the procedures as above for the malarial retinopathy images.

All the images were segmented using the framework proposed and the results were compared and evaluated against the manual delineation. The framework is implemented in Matlab 7.12.0 (R2011a) on a 32-bit operating system (Intel(R) Core(TM) i3-2100).

### B. Evaluation Metrics

Three commonly used metrics were used to evaluate performance: sensitivity, specificity and accuracy in terms of pixels. Sensitivity (resp. specificity) is a measure of the effectiveness in identifying positive (resp. negative) pixels, while accuracy is a metric to indicate the overall classification performance. These metrics are defined as follows:
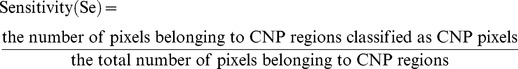


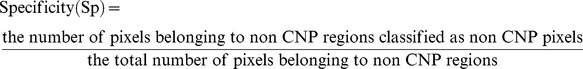


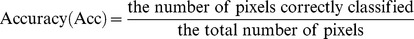
In addition, the overlapping ratio is used as an extra performance measure to evaluate the segmentation. The overlapping ratio marks the number of regions that coincide with reference standard regions. It is possible for the regions segmented from the proposed framework to overlap all regions identified by the expert without sharing identical boundaries. For this reason a combination of overlapping ratio and pixel-by-pixel performance measurement can provide a more comprehensive understanding of the segmentation performance. Following the method Buchannan and Trucco et al.'s method [40], the region overlapping ratio was interpreted as follows: region classified as a CNP and overlaps with the expert's annotation over the total number of regions in expert's annotation.

### C. Parameter Sensitivity Test

In order to demonstrate robustness of the kernel mutual information model to parameter variation, effects of region weighting factor (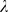) in the energy minimization model were evaluated. 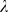 controls the balance between smoothness of the detected boundary and the uniformity of detected regions. A smaller value of 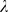 provides a smoother region boundary, while a larger 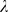 provides a more complex and more sensitive boundary. In this test, a range of values are presented to show sensitivity of this framework with different values for 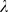. In addition, the elapsed time of each computation is measured using the MATLAB ‘tic toc’ function for comparing the relative speed of the algorithm.

In the case of supervised region-wise refinement, the cost coefficient and number of learners are the two parameters that are optimized. The cost coefficient controls the weight for penalizing false positives, where the number of learners is the number of decision trees used to build an ensemble classifier. Both parameters were tested in combination to obtain the best classifier. The classifier is evaluated through a five-fold cross validation where the dataset was divided into five folds, and each fold will in turn be kept for testing while the other four folds were used for training the AdaBoost classifier. In order to test the robustness of classifiers, five-fold cross-validation was repeated five times - each time with random permutation to divide the data.

## Results

### A. Results from Images of Malarial Retinopathy

#### 1) Results from Unsupervised Segmentation

**Figure 5:**
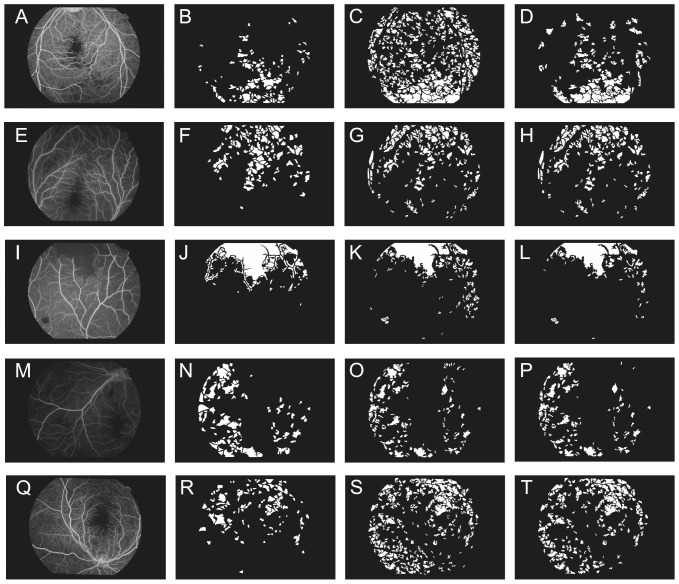
Illustration of segmentation results of five malarial retinopathy images. Each row represents a case (left) and its manual delineation (middle left), intermediate segmentation results after step 2 – unsupervised segmentation (middle right), and step 3 – supervised segmentation (right).

The variational texture segmentation algorithm that we have developed will stop when either the number of iterations reaches a maximum of 2000 or the difference between two consecutive iterations is less than 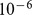. Table 1 reports how a range of the parameter 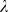 can affect segmentation in terms of pixel by pixel accuracy, sensitivity and specificity, and time efficiency. From this table, it can be seen that segmentation performances are consistent, but computational time varies. For this unsupervised step 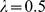 was chosen for our analysis, as it yields the best sensitivity. Fig. 5 illustrates the segmentation of some example images results pre and post refinement. After unsupervised segmentation, across the collection, we obtained on average a pixel by pixel accuracy of 87.1%, sensitivity of 74.9% and specificity of 88.7%. Using the aforementioned region overlap agreement, we report an average ischemic region detection of 79.7% (47/59) (number of regions in reference standard detected/number of regions in reference standard) and median false positive of 164 with range of 19–264.

**Table 1:** Results of parameter sensitivity test for step 2 – unsupervised segmentation for the malarial retinopathy dataset.

*_λ_*	*Accuracy*	*Sensitivity*	*Specificity*	*Elapsed Time (seconds)*
**0.3**	0.871±0.048	0.749±0.144	0.888±0.063	371.2±372.2
**0.5**	0.871±0.048	0.749±0.144	0.887±0.063	266.3±264.2
**1**	0.871±0.048	0.748±0.144	0.888±0.063	199.4±47.8
**1.5**	0.871±0.048	0.748±0.144	0.888±0.063	192.6±14.5
**2**	0.871±0.048	0.748±0.144	0.888±0.063	190.1±7.6

Including overall mean±standard deviation of pixel by pixel accuracy, sensitivity, specificity, and elapsed segmentation time from using various regularization weighting factor 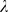.

#### 2) Final Results after Region-wise Refinement

**Figure 6:**
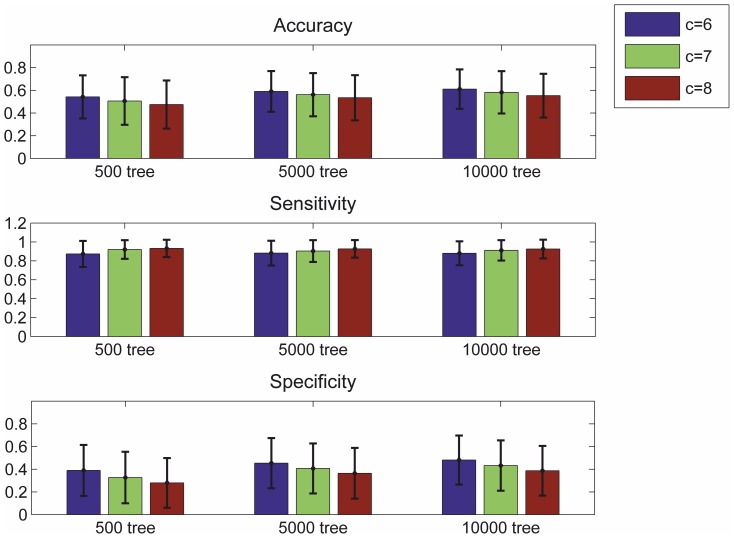
Region-wise evaluation of the supervised ensemble classifier used in 2nd segmentation. Parameters of the ensemble classifier: number of trees and the weight value were tested in different combinations. Columns from left to right are c = 6, 7 & 8 of each ensemble classifiers with 500, 5000 and 10,000 trees. This figure shows region-wise accuracy, sensitivity and specificity, obtained from the average of five tests with different seed points for a five-fold cross-validation in each test.

The effect of varying number of trees and cost coefficient values were tested as described above, five-fold cross validation was performed with different partitions. The range tested include 500, 1000, 2000, 5000 and 10,000 trees, in combination with cost coefficients of 5 to 9. The 5000 tree learner with a cost coefficient of 8 was found to be the most effective combination (Fig. 6). With this set of parameters for the supervised step we were able to achieve a pixel by pixel accuracy of 89.0%, sensitivity of 73.0% and specificity of 90.8%. The average region overlapping ratio after refinement is 76.3% (45/59). The median number of false positives is 86 (range: 17 to 166). See Table 2 for the summary of results from step 2 and 3.

**Table 2:** Summary of results from step 2 and 3 (unsupervised and supervised respectively) segmentation on the malarial retinopathy dataset, presented in region-wise and pixel-wise performance metrics.

	Average Regions Overlapped	Median Region False Positives (Range)	Pixel-wise Accuracy	Pixel-wise Sensitivity	Pixel-wise Specificity
**Step 2**	47/59	164 (19–264)	0.871±0.048	0.749±0.144	0.887±0.063
**Step 3**	45/59	86 (17–166)	0.890±0.047	0.730±0.142	0.908±0.059

### B. Results from Images of Diabetic Retinopathy

The segmentation framework was applied to the ten diabetic retinopathy images following the same evaluation strategy as above for the malarial retinopathy images. 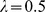 was chosen for the unsupervised segmentation step. The effect of varying number of trees and cost coefficient values were tested as described above, five-fold cross validation was performed with different partitions. The range tested included 500, 1000, 2000, 5000 and 10,000 trees, in combination with cost coefficients of 1 to 19 with an interval of 2. The 500 tree learner with a cost coefficient of 19 was found to be the most effective combination. With this setting for the supervised step we were able to achieve a pixel by pixel accuracy of 80.8%, sensitivity of 72.6% and specificity of 82.1%. The average region overlapping ratio after refinement is 73.9% (17/26).

## Discussion & Conclusions

We have designed a novel texture segmentation framework to tackle the CNP segmentation problem in retinal angiography. The framework comprises three major steps: pre-processing, total variation texture segmentation, and supervised segmentation. The first highlight of this framework is the state-of-the-art multiphase total variation texture segmentation model and a new kernel based region term, providing an elegant way to segment texture. The second highlight is the use of supervised segmentation to incorporate expert knowledge to guide refinement in order to achieve a more application specific result. One significant challenge in using supervised classification is dealing with imbalanced data. In the specific application of CNP, the number of CNP and non-CNP are skewed. A weighted strategy appears more appropriate to penalize misclassification errors for each class differently. Weighted Adaboost was chosen for our specific application for its simplicity and efficiency, but other methods such as weighted-SVM [41] can be easily adapted to this framework to extend its range of applications.

To demonstrate the functionality of the proposed framework, we applied it to retinal images of CNP arising from two distinct etiologies, which were acquired using different imaging modalities – malarial retinopathy (conventional FFA) and diabetic retinopathy (scanning laser ophthalmoscope). The segmentation framework demonstrated satisfactory performance: accuracy of 89.0%, sensitivity of 73.0%, and specificity of 90.8% on forty-eight images from malarial retinopathy, and accuracy of 80.8%, sensitivity of 70.6%, and specificity of 82.1% on ten diabetic maculopathy images. In terms of region-wise analysis, the framework achieved an accuracy of 76.3% (45/59) regions for the malarial retinopathy images and 73.9% (17/26) for the diabetic maculopathy images, respectively. To the best of our knowledge, this is the first study that has used such a large collection of FFA images to address this challenging problem. Our experimental results provide an original insight to this medical imaging problem. Calculations of the percentage of CNP and their distributions may have utility in determining clinical progression of disease and associations with systemic complications.

Automated analysis of retinal images is an important topic of research [11], [42]. The main emphasis has been on analysis of color fundus photographs rather than FFA, and so the problem of detecting CNP is relatively unexplored. Jasiobedzi et al. was the first group to report non-perfusion detection in angiographic retinal images [12], [13]. This group stressed the importance of utilizing texture data in FFA and captured them using extensive morphological operations and region merging. They found that merged regions yielded better results than smaller regions, but did not report sensitivity, specificity or accuracy [12]. Sivaswamy et al. reported an unsupervised CNP segmentation method using a variance-based region growing technique [43]. They evaluated their method on 40 FFA images and reported an area under curve (AUC) of 0.842 using pixel by pixel evaluation. Their ROC suggests a sensitivity of 0.9 and specificity of 0.36. Trucco et al. used temporal and contextual information to classify ischemic areas and capillary leakage in ultra-wide field of view (UWFV) FFA sequences to maximize area of coverage [40], [44]. Five FFA sequences were analyzed in [44] and an accuracy of about 80% was reported in terms of pixel-wise measure. 16 sets of sequential images were analyzed [40], and they evaluated their technique in terms of region overlapping, which is significant to CNP segmentation. This type of assessment is important because ground-truth CNP boundaries are extremely subjective, and a ROC statistics may not be an appropriate measure of accuracy. From their evaluation 71.5% (93/130) of regions were correctly located but with over 80 false positives (no maximal or mean value of the number of false positive numbers is reported). Their method is based on UWFV image sequences which have more frames and each frame has much wider field than images of this study, hence it is not possible to compare the performance directly. Nevertheless, our segmentation framework will be applicable to UWFV images after certain level of adaption and optimization.

It is a challenging task to achieve very high detection performance for automatic segmentation of CNP in FFA images. From our experience and others, there are many different factors that could compromise the performance. First of all, there is a very large variation in terms of appearance (e.g. brightness, contrast, and artefact) across images, this makes it difficult to have universal criteria to define CNP. That is, a region with similar appearance may be CNP in one image but not in other images. Secondly, there are many confounders within an image. For example, hemorrhage often appears in images and has similar view to CNP (see row 3 and 4, Fig. 5). Regions of hemorrhage will cause problems in training the classifier and result in low accuracy (some CNP will be misclassified as hemorrhage or hemorrhage as CNP). It would be ideal to develop a program to remove them before CNP detection in the future. The appearance of CNP in an image may vary due to uneven illumination and other artefacts. For example, CNPs in the center of an image often look different to those at the edge of the FOV, and regions close to the edge of the FOV may mimic as CNP due to poorer focusing problem as the retina is a curved surface. Future development should take into account of these issues. On the other hand, recently there is a trend in development of interactive segmentation programs to address challenging segmentation problems. An interactive segmentation strategy may be adopted to address the CNP segmentation problem for higher performance. For example, following [45] we can use an unsupervised segmentation program to first detect candidate CNP regions and leave potential users to select areas of true CNP. The programs will report the quantitative information of CNPs after user selection, potentially providing good performance with minimal human involvement.

Texture is an effective feature for CNP segmentation, however manual grading involves the whole FA sequence, and therefore incorporates additional contextual and temporal information. As reported by Trucco et al. [40], [44] it is important to exploit the evolution of intensities over time and incorporate contextual knowledge of pathology into automated segmentation. Texture has proved to be a useful feature, but in isolation is not comprehensive enough. Therefore in future studies, data between the early venous and late venous phases of FFA will be included as temporal and contextual features. In addition the FOV can be enlarged by overlapping different FOV to create a montage, with the aim of gathering data from the peripheral retina as well as the posterior pole. Computational time is reasonable (approx. 5 to 6 minutes for 3008×1960 pixels), but can be increased by using C++ or graphic processor unit (GPU) techniques. In this study we have used a single expert as the reference standard, and plan to improve on this by evaluating the inter- and intra-observer variation of manual CNP segmentation prior to future clinical application.

Development of this framework is motivated by medical demands for a tool to measure regions of retinal CNP in FFA images of the eye. Although designed with retinal imaging in mind, this framework can be applied to images from diverse imaging modalities in healthcare, including ultrasound, CT, and MRI. It can also be applied to other more general image segmentation problems. Moreover, the flexibility of this framework also extends to addressing multiphase intensity and texture problems. This can be done by simply modifying the region term in the second step of the framework and by using multi-class classification in final step. Therefore we expect this framework to have wide clinical applications.

In conclusion, we report and evaluate a newly developed comprehensive segmentation framework to address to the problem of CNP region segmentation, and our experimental results demonstrate its effectiveness. The proposed framework combines the strength of unsupervised and supervised segmentation techniques. This framework has the potential to be further developed as a useful tool for fast, accurate and objective assessment of a wide range of retinal diseases. The flexibility of this framework will certainly make it applicable and valuable in other real world applications.
